# Molecular surveillance reveals the emergence and dissemination of NDM-5-producing *Escherichia coli* high-risk clones in Germany, 2013 to 2019

**DOI:** 10.2807/1560-7917.ES.2023.28.10.2200509

**Published:** 2023-03-09

**Authors:** Jörg B Hans, Niels Pfennigwerth, Bernd Neumann, Yvonne Pfeifer, Martin A Fischer, Jessica Eisfeld, Jennifer Schauer, Sebastian Haller, Tim Eckmanns, Sören Gatermann, Guido Werner

**Affiliations:** 1 National Reference Centre for Multidrug-resistant Gram-negative Bacteria, Department for Medical Microbiology, Ruhr University Bochum, Bochum, Germany; 2 Division of Nosocomial Pathogens and Antibiotic Resistance, Department of Infectious Diseases, Robert Koch-Institute, Wernigerode Branch, Wernigerode, Germany; 3 Institute for Hospital Hygiene, Medical Microbiology and Clinical Infectiology, Paracelsus Medical University, Nuremberg General Hospital, Nuremberg, Germany; 4 Landeszentrum Gesundheit Nordrhein-Westfalen, Fachgruppe Infektionsepidemiologie, Bochum, Germany; 5 Department for Infectious Disease Epidemiology, Robert Koch-Institute, Berlin, Germany

**Keywords:** Carbapenemase, Enterobacterales, Whole Genome Sequencing, WGS, One Health, E. coli, NDM-5, antibiotic resistance

## Abstract

**Background:**

Carbapenemase-producing Enterobacterales (CPE) are rapidly increasing worldwide, also in Europe. Although prevalence of CPE in Germany is comparatively low, the National Reference Centre for Multidrug-resistant Gram-negative Bacteria noted annually increasing numbers of NDM-5-producing *Escherichia coli* isolates.

**Aim:**

As part of our ongoing surveillance programme, we characterised NDM-5-producing *E. coli* isolates received between 2013 and 2019 using whole genome sequencing (WGS).

**Methods:**

From 329 identified NDM-5-producing *E. coli*, 224 isolates from known geographical locations were subjected to Illumina WGS. Analyses of 222 sequenced isolates included multilocus sequence typing (MLST), core genome (cg)MLST and single-nucleotide polymorphism (SNP)-based analyses.

**Results:**

Results of cgMLST revealed genetically distinct clusters for many of the 43 detected sequence types (ST), of which ST167, ST410, ST405 and ST361 predominated. The SNP-based phylogenetic analyses combined with geographical information identified sporadic cases of nosocomial transmission on a small spatial scale. However, we identified large clusters corresponding to clonal dissemination of ST167, ST410, ST405 and ST361 strains in consecutive years in different regions in Germany.

**Conclusion:**

Occurrence of NDM-5-producing *E. coli* rose in Germany, which was to a large extent due to the increased prevalence of isolates belonging to the international high-risk clones ST167, ST410, ST405 and ST361. Of particular concern is the supra-regional dissemination of these epidemic clones. Available information suggest community spread of NDM-5-producing *E. coli* in Germany, highlighting the importance of epidemiological investigation and an integrated surveillance system in the One Health framework.

**Key public health message**

**What did you want to address in this study?**

The emergence and spread of multidrug-resistant bacteria, in particular those not susceptible to last-resort antibiotics such as NDM-5-producing *Escherichia coli*, poses a threat to public health. We aimed to investigate the genomic relatedness of rapidly increasing NDM-5-producing *E. coli* in Germany.

**What have we learnt from this study?**

Whole genome sequencing has become an indispensable tool for the monitoring of emerging trends of antimicrobial resistance. The increased number of cases with NDM-5-producing *E. coli* is driven by local clusters and, more importantly, by the supra-regional spread of a few dominant clonal lineages.

**What are the implications of your findings for public health?**

NDM-5-producing *E. coli* appear to spread in the community through multiple sources and transmission routes, which highlights the need for intensified epidemiological investigations to address this public health threat.

## Introduction

Carbapenems are considered as last resort antibiotics used to treat patients with severe infections caused by multidrug-resistant Gram-negative bacteria. Because of their enzyme-mediated antibiotic resistance linked to high genetic mobility, carbapenemase-producing Enterobacterales (CPE) pose a major threat to public health. Over the past decades, CPE have rapidly spread, also in Europe, for which a worsened epidemiological situation has been reported [1]. Although prevalence of CPE in Germany is comparatively low, the National Reference Centre for Multidrug-resistant Gram-negative Bacteria (NRC) detected annually increasing numbers of CPE, specifically *Escherichia coli*. Besides the rapid spread of OXA-244 carbapenemase-producing *E. coli*, we have also observed an increase in *E. coli* isolates producing the New Delhi metallo-β-lactamase 5 (NDM-5) since 2017 [2,3].

NDM-5 is a variant of NDM-1 that differs by two amino acids (Val-88-Leu and Met-154-Leu) and has increased resistance to extended-spectrum cephalosporins and carbapenems. It was first described in 2011 in an *E. coli* isolate recovered from a patient in the United Kingdom (UK) with prior hospitalisation in India, which along with the Middle East and the Balkan region is considered the main reservoir of NDM producers [4,5]. Subsequently, NDM-5-producing Enterobacterales have rapidly spread worldwide with some countries facing an endemic situation, such as China [5,6]. In contrast, NDM-5-producing Enterobacterales have only sporadically been reported from Europe [7-9]. Although most of these cases were associated with international travel or hospitalisation, there have also been reports on patients who had not previously travelled, suggesting that NDM-5-producing *E. coli* were community-acquired [10-12]. Of further concern, NDM-5-producing Enterobacterales have been recovered from a variety of other sources worldwide, including food, livestock, companion animals, wildlife and the environment [13-18]. Furthermore, the *bla*_NDM-5_ gene has been found on different plasmid types, thus enhancing its dissemination capability [6,12-14]. However, unlike NDM-1, which exhibits a wide host range, NDM-5 has primarily been identified in *E. coli* and recent studies implicate that certain epidemic clones may also contribute to its increased emergence and spread [19-22]. However, these studies analysed only a limited number of isolates from a short time period but more data are needed to understand the genomic epidemiology of NDM-5-producing *E. coli* in Europe.

As part of our ongoing molecular surveillance programme, we here provide a detailed investigation of NDM-5-producing *E. coli* in Germany. We used whole genome sequencing (WGS) to characterise NDM-5-producing *E. coli* isolates circulating in Germany between 2013 and 2019.

## Methods

### Setting and data source

Since 2009, the NRC has been located in the Department for Medical Microbiology at the Ruhr-University Bochum in Bochum, Germany. For German primary diagnostic laboratories, both clinical and private, it exclusively provides the free service for the verification and genotyping of suspected carbapenemase-producing isolates. These laboratories are requested, but not obliged, to send Enterobacterales isolates that fulfil specific criteria, for *E. coli* these are: elevated minimum inhibitory concentrations (MIC) for ertapenem (> 0.5 mg/L), meropenem or imipenem (> 2 mg/L) or decreased inhibition zone diameters of < 25 mm for ertapenem (10 µg) or < 25 mm for meropenem (10 µg) or imipenem (10 µg), largely following the European Committee on Antimicrobial Susceptibility Testing (EUCAST) guidelines [23]. Along with the sample, diagnostic laboratories are asked to provide basic epidemiological data by filling in a structured submission form in accordance with the German data protection law, including information on the patients’ sex, date of birth, inpatient or outpatient status, geographical location as based on first three numbers of the German five-digit postal code referring to the hospital or surgery where the isolate was sampled, isolate source, infection status and information on prior hospitalisation or stay abroad 6 months before detection.

Details on phenotypic and molecular methods used at the NRC for the identification of carbapenemases are described elsewhere [24]. In brief, a comprehensive range of phenotypic tests is used to detect Enterobacterales isolates suggestive of being carbapenemase-producing. Individual PCR amplifications of KPC-, VIM-, IMP-, NDM- and OXA-48-encoding genes followed by the sequencing of PCR amplicons are routinely used to confirm and identify the carbapenemase genes.

### Samples and whole genome sequencing

Between 2013 and 2019, the NRC identified 329 non-duplicate NDM-5-producing *E. coli* isolates, including 224 isolates of known geographical location, which were obtained from 224 single patients. For these 224 NDM-5-producing *E. coli* isolates, bacterial cultures were grown overnight in Luria-Bertani (LB) medium (PanReac AppliChem ITW Reagents, Darmstadt, Germany) supplemented with meropenem (2 mg/L). Total genomic DNA from 1 mL bacterial cultures was extracted (Easy-DNA gDNA Purification Kit, Invitrogen, Thermo Fisher Scientific, Schwerte, Germany) and prepared (Nextera XT DNA Library Preparation Kit, Illumina, Eindhoven, the Netherlands) for WGS. Libraries were paired-end sequenced on a HiSeq 1500 (2 × 251 bp) or NextSeq (2 × 151 bp) instrument (Illumina, San Diego, United States) and quality of raw sequence data was checked using FastQC v0.11.9 [25]. Mash Distance v2.1 and Mash Screen v2.1 were performed on raw reads for species identification and contamination check, respectively [26,27]. Reads were de novo assembled using SPAdes v3.10.1 with default parameters in the careful mode [28]. Quality metrics of assemblies were assessed using QUAST v5.0.2 without a reference sequence [29]. This dataset also included raw data of two isolates co-harbouring *bla*_NDM-5_, which were sequenced as part of a previous study [3].

### Phylogenetic and in silico analyses

Followed by in silico screening of resistance genes using ABRicate [30] and the National Center for Biotechnology Information (NCBI) Antimicrobial Resistance Gene Finder Plus database [31], assemblies passing quality controls were uploaded to the SeqSphere+ software version 7.7.5 (Ridom, Muenster, Germany), which was used for multilocus sequence typing (MLST) following the Achtman scheme and core genome (cg)MLST based on the Enterobase *E. coli* scheme (2,513 loci). Pairwise allelic differences between isolates were used to construct a neighbour-joining tree with metadata annotated using Interactive Tree Of Life (iTOL) version 6.5.7 [32]. We further conducted single-nucleotide polymorphism (SNP)-based analyses of the core genome for isolates of specific sequence types (ST). Using the CSI Phylogeny 1.4 server (Call SNPs & Infer Phylogeny; https://cge.cbs.dtu.dk/services/CSIPhylogeny) with default settings, which includes the pruning of SNP within 10 bp, a separate phylogenetic analysis was performed on assemblies of isolates of each ST to construct an SNP distance matrix that was visualised in a heatmap using iTOL. Clusters within the phylogenetic tree corresponding to clonal dissemination were defined based on a pairwise SNP distance of ≤ 100 between isolates, as has recently been suggested for OXA-244-producing *E. coli* in France [33]. In this context, it has to be noted that, in the absence of a clear consensus on the maximum cut-off to define clonality, the SNP distance chosen here may appear high compared with thresholds typically used to elucidate local outbreaks. However, to account for our long-term surveillance data, we used this liberal threshold as we expect that identified clusters may reflect patterns on a broader genetic scale, such as clonal lineages. Moreover, we did not correct for recombination which is known to increase genetic diversity. Therefore, we are confident that the SNP distances obtained represent a reliable measure of genetic relatedness between isolates. To illustrate distribution patterns, cluster isolates were mapped based on the first three digits of the postal code.

## Results

### Epidemiology of NDM-5-producing *Escherichia coli*

**Figure 1:**
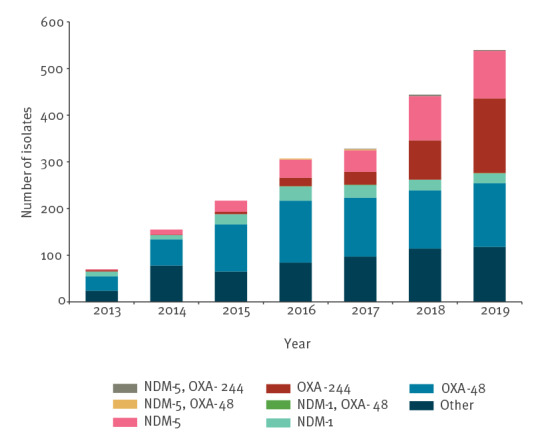
Carbapenemase-producing *Escherichia coli*, Germany, 2013–2019 (n = 2,061)

Since 2013, we have observed annually increasing numbers of NDM-5 and OXA-244-producing *E. coli* isolates, whereas the occurrence of other carbapenemases in *E. coli* including OXA-48, the most prevalent carbapenemase in Enterobacterales in Germany, has remained comparatively constant since 2016 (Figure 1). Numbers of NDM-5-producing *E. coli* isolates increased from two isolates sent by two diagnostic laboratories in 2013 to 11 from eight laboratories in 2014 and to 24 from 19 laboratories in 2015, whereas 41 and 49 isolates were obtained from 33 diagnostic laboratories in 2016 and 2017, respectively. Ninety-eight isolates were submitted from 64 diagnostic laboratories in 2018 and 104 isolates from 68 diagnostic laboratories in 2019, the 2 years with the highest increase in NDM-5-producing *E. coli*. Taken together, annual increases of NDM-5-producing *E. coli* were paralleled by an increasing number of laboratories submitting isolates, which indicates that the rise in detection was not due to locally restricted outbreaks. Until 2019, NDM-5 had become the third most prevalent carbapenemase among *E. coli* isolates in Germany (Figure 1).

**Figure 2:**
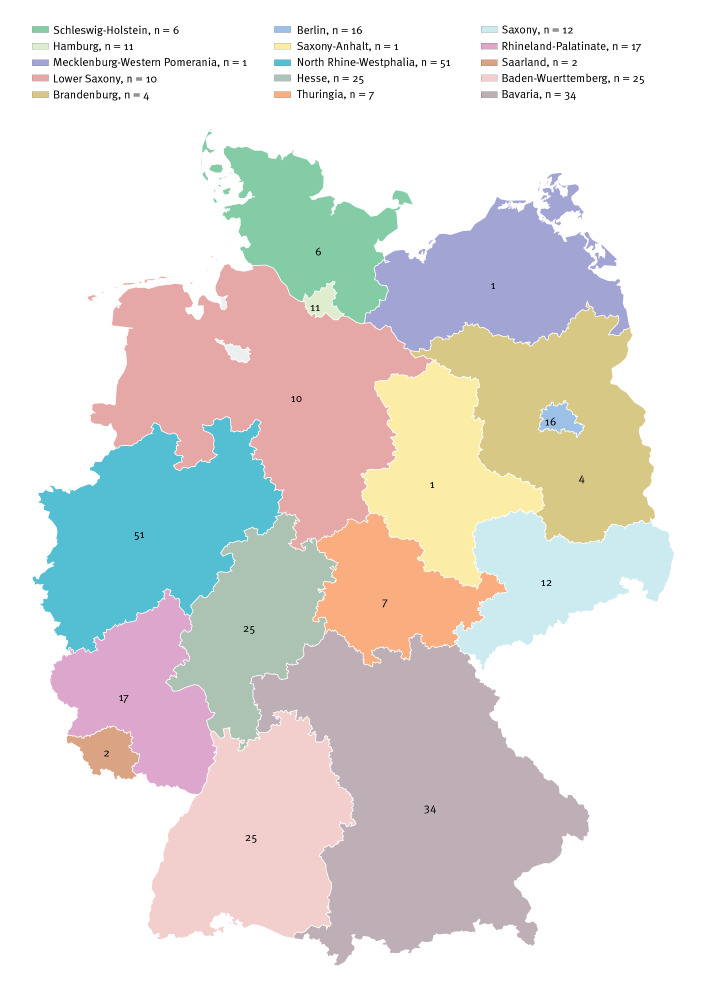
NDM-5-producing *Escherichia coli* isolates by federal state, Germany, 2013–2019 (n = 222)

Of the 224 NDM-5-producing *E. coli* sequenced, genomic data of 222 isolates passed quality controls and were included in further analyses. Submitted from a total of 98 different diagnostic laboratories, the 222 NDM-5-producing *E. coli* isolates were obtained from hospitals or surgeries in 15 of the 16 German federal states with numbers ranging from 1 to 51 isolates per federal state (Figure 2). Almost half of the isolates (110/222; 49.5%) were obtained from North Rhine Westphalia (n = 51), Bavaria (n = 34) and Baden-Wuerttemberg (n = 25), which are the regions with the largest populations in Germany. At the time of sampling, most patients were hospitalised: 94 patients were on a general ward, 34 patients were in an intensive care unit, 25 isolates were obtained from ambulatory patients, while for the remaining 69 cases, no information was given by the primary diagnostic laboratory. 

The ratio of the patients’ sex was almost equally distributed with 99 males and 85 females, while for 38 patients, the sex was not stated. For 221 of the 222 cases, the age was known, ranging from 1 to 94 years (median: 62 years). The majority of the 222 NDM-5-producing *E. coli* isolates were obtained from screening samples (116/222; 52.2%) followed by urine samples (66/222; 29.7%). The remaining isolates were from wound infections (12/222; 5.4%), other screening samples (9/222; 4.1%), blood cultures (4/222; 1.8%), intra-abdominal locations (4/222; 1.8%), respiratory tract (4/222; 1.8%), skin (2/222; 0.9%), other sources (2/222; 0.9%) and three isolates were of unknown origin. In accordance, patients were more often colonised (79/222; 35.6%) than infected (37/222; 16.7%), although this information was lacking for about half of the cases (106/222; 47.7%). Metadata for each isolate are provided in Supplementary Table S1.

### Phylogenetic cgMLST and in silico analyses

**Figure 3:**
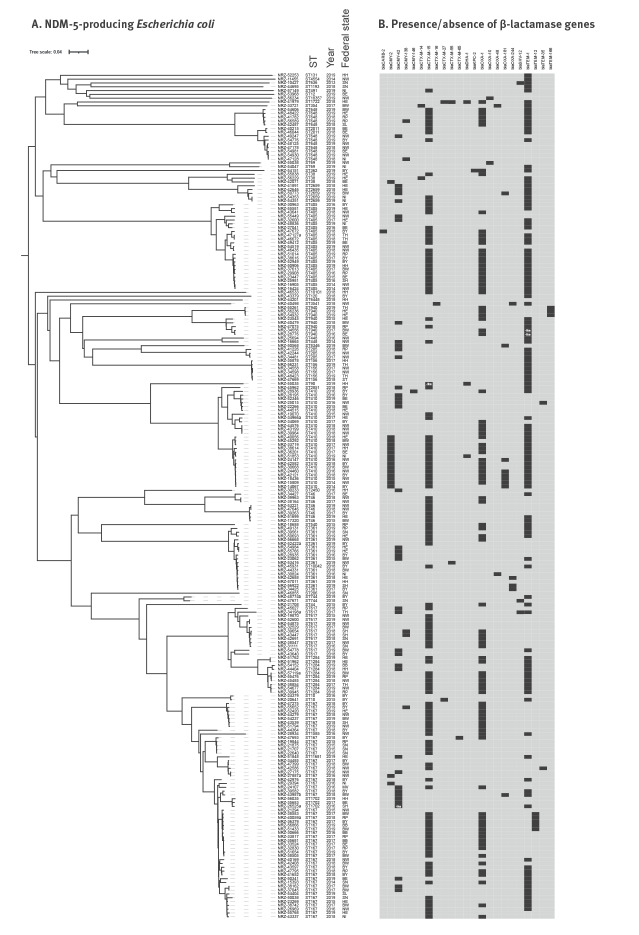
Clustering and gene content of NDM-5-producing *Escherichia coli* isolates, Germany, 2013–2019 (n = 222)

The 222 isolates of NDM-5-producing *E. coli* belonged to 43 different ST. Most prevalent were ST167 (n = 52), ST410 (n = 26), ST405 (n = 23) and ST361 (n = 16) accounting for more than half of the isolates (52.7%), while a total of 24 different ST were represented only by a single isolate (Figure 3). The cgMLST analysis revealed patterns of close genetic relatedness among isolates for many of the different ST (Figure 3). In particular, distinct clusters were identified among isolates belonging to ST167, ST410, ST405 and ST361, together with ST10042 (a single-allele derivative of ST361). Indeed, similar resistance gene patterns were found for most of the clustered isolates of ST167, ST410, ST405 and ST361/10042, further supporting their close genetic relatedness (Figure 3).

Panel A: Phylogenetic tree of NDM-5-producing *Escherichia coli* including the ST, the year and federal state shown for the isolates. Panel B: Gene matrix showing the presence (black) or absence (light grey) of β-lactamase genes, respectively. Incomplete gene sequences are denoted “-like”.

Multiple resistance genes were identified by in silico analyses among the 222 isolates of NDM-5-producing *E. coli* (Figure 3B). Additional carbapenemase-encoding genes (*bla*_KPC-2_, *bla*_OXA-48_, *bla*_OXA-181_ or *bla*_OXA-244_) were carried by 16 of the 222 isolates. Additional extended-spectrum β-lactamase (ESBL)-encoding genes (*bla*_CTX-M-14_, *bla*_CTX-M-15_, *bla*_CTX-M-16_, *bla*_CTX-M-27_, *bla*_CTX-M-55_, *bla*_CTX-M-65_, *bla*_SHV-12_, *bla*_TEM-1_, *bla*_TEM-12_, *bla*_TEM-35_ and *bla*_TEM-166_) were identified in 181 isolates and plasmid-encoded *ampC* genes (*bla*_CMY-2_, *bla*_CMY-42_, *bla*_CMY-138_ or *bla*_CMY-146_) were found in 61 isolates, whereas four isolates contained incomplete sequences of single β-lactamase genes (Figure 3B). The *bla*_NDM-5_ gene was generally found on short contigs and it was therefore not possible to determine its plasmid or chromosomal location.

### Phylogenetic SNP analyses and mapping of cases

**Figure 4:**
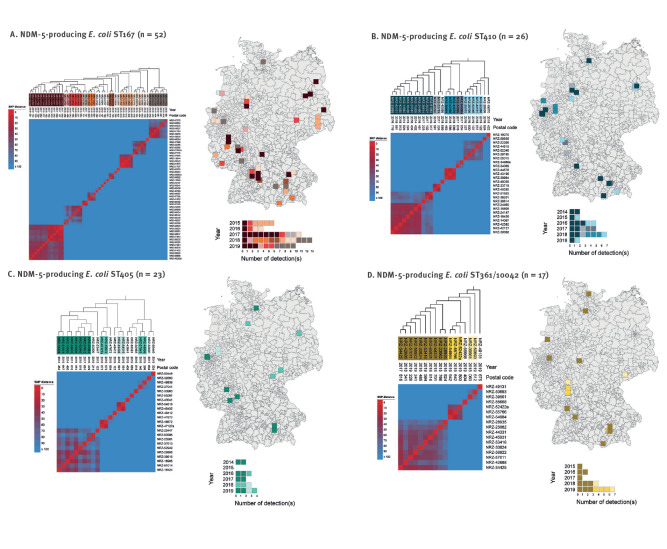
Heatmaps of pairwise SNP differences between NDM-5-producing *Escherichia coli* isolates as well as spatial and temporal distribution of strains, Germany, 2013–2019

To further examine the genetic relatedness of isolates, we performed separate core genome SNP analyses, which revealed large clusters among isolates of each ST corresponding to clonal dissemination of strains as follows: 13 of 52 ST167 isolates (1–61 SNP distance, median: 41 SNP) obtained from nine regions between 2015 and 2019, 11 of 26 ST410 isolates (18–94 SNP distance, median: 40 SNP) obtained from eight regions between 2014 and 2019, eight of 23 ST405 isolates (29–89 SNP distance, median: 59 SNP) obtained from six regions in 2014, 2016, 2017 and 2019, and 10 of 17 ST361/10042 isolates (28–89 SNP distance, median: 57 SNP) obtained from nine regions between 2015 and 2019 (Figure 4). Although isolates received from the same or a neighbouring three-digit postal code area suggest that sporadic regional transmissions were likely, overall mapping of cluster isolates of ST167, ST410, ST405 and ST361/10042 revealed supra-regional distribution patterns. In contrast, isolates from the smaller clusters were generally obtained from the same area, indicating possible regional spread (Figure 4).

SNP: single nucleotide polymorphism.

Colours in the heatmap represent SNP numbers as shown in the legend on the left. Phylogenetic trees including the year and the three-digit postal code for the isolates are shown on top of the heatmap. Isolates from clonal clusters are colour-coded and mapped based on the three-digit postal code as well as shown by annual number of detections.

## Discussion

We here provide further evidence for the rapid and ongoing spread of CPE in Germany. Previously, we reported on the increase in OXA-244-producing *E. coli*, predominantly driven by genetically clustered isolates of ST38, that was also observed in at least nine other European countries [3]. In contrast, increasing numbers of NDM-5-producing *E. coli* have thus far only been communicated from a few European countries. Since 2012, a constant increase in the number of NDM-producing Enterobacterales has been observed in France, albeit not specifically NDM-5-producing *E. coli* [34]. Similarly, Switzerland has observed a rise in detections of NDM-producing Enterobacterales since 2013 [35] and recently reported an increased prevalence of NDM-producers, including NDM-5-producing *E. coli* for which successful global lineages have been attributed as the major cause [22]. Likewise, we showed that the increase in NDM-5-producing *E. coli* in Germany was to a large extent due to isolates belonging to ST167, ST410, ST405 and ST361, all of which are regarded as high-risk clones ([19,22], and references therein). We further showed through phylogeographical analyses the clonal dissemination of certain ST167, ST410, ST405 and ST361/10042 strains on a supra-regional scale. Similar results were obtained from a collaborative study between Germany and Switzerland, which also identified ST167, ST405, ST361, but not ST410, to be prevalent among a series of NDM-5-producing *E. coli* isolates including strains found in both countries, indicating cross-border spread [21]. Thus, there is increasing evidence of the emergence and polyclonal dissemination of NDM-5-producing *E. coli* high-risk clones, at least in Germany and Switzerland.

However, cases with ST167 and ST405 are also increasingly reported from Italy, where an autochthonous hidden reservoir of NDM-5-producing *E. coli* has been proposed [20,36-39]. Indeed, although we cannot exclude the possibility of travel-associated transmissions from endemic regions, it is unlikely that the observed increase in NDM-5-producing *E. coli* in Germany was due to elevated importation of cases. In such a situation, one would expect that other European countries and not only those mentioned may have experienced an increase in NDM-5-producing *E. coli*. Furthermore, overall distribution patterns of NDM-5-producing *E. coli* strains cannot be explained by sustained nosocomial transmissions on a supra-regional scale despite certain instances of sporadic healthcare-associated infections. In this context, however, it has to be noted that, due to the lack of detailed epidemiological data, we cannot exclude the possibility that prior exposure to healthcare settings other than hospitals might play a role in the transmission of NDM-5-producing *E. coli* in Germany. Furthermore, it has to be mentioned that we do not know the exact number of hospitals in which these strains might circulate because several hospitals can be located within the same district covered by the three postal code digits. Available data rather suggest that NDM-5-producing *E. coli* were acquired outside the hospital setting because the majority of isolates were obtained from screening samples before or accompanying hospital admission. However, because the majority of isolates were found in a nosocomial context, these cases might be representative neither for the place of acquisition nor for the underlying mode of transmission of NDM-5-producing *E. coli* in Germany. For a more detailed investigation and identification of sources of NDM-5-producing *E. coli*, more clinical and epidemiological metadata are required, which cannot be provided as part of NRC routine work and have to be addressed in targeted surveillance activities with support from public health authorities.

NDM-5-producing *E. coli* ST167, ST405 and ST410 have also been detected in dogs in Italy, Switzerland and the UK and a confirmed transmission in Finland between humans and dogs or vice versa [15,40-42]. Furthermore, NDM-5-producing *E. coli* ST167 and ST410 have also been identified in river samples in Switzerland indicating their dissemination in the environment [18]. In Germany, widespread distribution of clonal *E. coli* ST410 associated with CTX-M-15 has already been observed among humans, animals and food products [43,44]. In this context, it has previously been shown that a specific *bla*_CTX-M-15_-carrying lineage of ST410 recently acquired the carbapenemases *bla*_OXA-181_ and *bla*_NDM-5_, as identified for some of our isolates [45]. Thus, we speculate that certain ST410 strains from this study might represent this newly emerged lineage which has already been widespread in Germany though not yet described with carbapenemases. In Greece, an NDM-5-producing *E. coli* ST361 isolate has been identified in a bovine sample [46]. Thus, multiple sources and transmission routes can be hypothesised regarding the reservoirs and spread of NDM-5-producing *E. coli* isolates and *bla*_NDM-5_-carrying genetic elements, clearly pointing towards the necessity of an integrated, One Health-oriented molecular surveillance approach.

## Conclusion

We here provide a detailed genomic analysis of NDM-5-producing *E. coli* isolates in Germany. We further demonstrate the usefulness and importance of a WGS-based approach for monitoring and resolving emerging resistance trends. Overall, it has to be noted that a lack of relevant metadata substantially limits conclusions drawn from such investigations. We thus propose that detailed epidemiological data should be routinely collected, assessed and provided as an essential part of a comprehensive and integrative genomic surveillance approach. In addition, our findings reinforce the need for cross-border data sharing and intersectoral collaborations to better elucidate this public health crisis and to streamline and optimise infection prevention and control measures.

## Supplementary Data

39000 Supplement
